# Development of a Novel SPR Assay to Study CXCR4–Ligand Interactions

**DOI:** 10.3390/bios10100150

**Published:** 2020-10-21

**Authors:** Arnaud Boonen, Abhimanyu K. Singh, Anneleen Van Hout, Kalyan Das, Tom Van Loy, Sam Noppen, Dominique Schols

**Affiliations:** Laboratory of Virology and Chemotherapy, Department of Microbiology, Immunology and Transplantation, Rega Institute, KU Leuven, Herestraat 49, P.O. Box 1030, 3000 Leuven, Belgium; abhi.singh@kuleuven.be (A.K.S.); anneleen.vanhout@kuleuven.be (A.V.H.); kalyan.das@kuleuven.be (K.D.); tom.vanloy@kuleuven.be (T.V.L.); sam.noppen@kuleuven.be (S.N.); dominique.schols@kuleuven.be (D.S.)

**Keywords:** surface plasmon resonance, G protein-coupled receptors, CXCR4, histidine tag, nanobodies, kinetics

## Abstract

G protein-coupled receptors (GPCRs) are involved in a plethora of different diseases. Consequently, these proteins are considered as an important class of drug targets. Measuring detailed kinetic information on these types of proteins has been challenging. Surface plasmon resonance (SPR) can provide this information, however, the use of SPR on GPCRs remains a complex issue. Here, we report an SPR assay to investigate the interactions between the full-length chemokine receptor CXCR4 and nanobody-Fc (Nb-Fc) ligands. Nb-Fcs consist of two monovalent VHH domains fused with an Fc domain of a human IgG molecule. The CXCR4 protein used in this assay was produced with a C-terminal 10x-histidine tag and was immobilized on a nitrilotriacetic acid chip. In order to verify the sensitivity and effectiveness of this assay, the results were compared to data obtained from cellular assays as well as from another SPR assay using CXCR4 virus-like particles (VLPs). CXCR4 remained intact and stable for at least 12 h, and the kinetic results correlated well with both the cellular assays and the VLP SPR assay results. Apart from determining the binding kinetics of Nb-Fc with CXCR4, our results contributed to understanding CXCR4 interaction dynamics. In conclusion, this assay provides a viable experimental platform that has high potential to be expanded for studying other molecules as well as other histidine-tagged GPCRs.

## 1. Introduction

G protein-coupled receptors (GPCRs) are cell membrane receptors representing a very important class of drug targets. Currently, about 34% of FDA-approved drugs target GPCRs [1]. Due to their therapeutic significance, GPCR research is a major subject in medical research and drug discovery. The CXC chemokine receptor 4 (CXCR4) is a GPCR that interacts with the CXC motif chemokine 12 (CXCL12), its natural ligand. Several isoforms of CXCR4 exist, which differ in the amino acid composition of their N-termini [2,3]. CXCR4 has a calculated molecular weight of ~40 kDa, which can vary due to different glycosylation patterns and post-translational modifications [4]. CXCR4 is widely expressed on many hematopoietic cell types, endothelial cells and stem cells, and its expression is highly pronounced in the bone marrow and lymphoid tissues [5]. Studies with genetic knockouts of both CXCR4 and CXCL12 underscored the vital role of this signaling axis during embryogenesis [6,7,8]. Apart from its role in normal physiology and development (including hematopoiesis, organogenesis, leukocyte migration and development of hematopoietic, cardiovascular and neuronal systems), CXCR4 is also involved in several pathologies. For instance, CXCR4 is highly expressed on many types of tumor cells allowing them to hijack CXCL12-CXCR4 pathway for metastasis of tumor cells to distinct organ sites where CXCL12 is highly expressed. It also functions as a major co-receptor for HIV entry in target cells [9]. The majority of developed CXCR4-targeting ligands are small molecules, primarily due to the advantage of low development cost and a lower potential for immunogenic responses. Another interesting class of molecules is monoclonal antibodies due to their high specificity and longer half-life [10,11]. Nanobodies (Nbs), closely related to antibodies, are derived from the variable antigen binding domain (VHH) of heavy chain antibodies (HCAbs), found in the Camelidae family’s immune system [12,13]. Recently, we reported a series of monovalent Nbs targeting CXCR4 that show CXCR4 binding affinity, impede the binding of CXCL12 to the receptor, inhibit CXCL12-induced calcium mobilization and demonstrate in vitro antiviral activity against HIV-1 [14]. These monovalent Nbs were also modified into bivalent nanobody-Fc (Nb-Fc) constructs, consisting of two VHH domains fused with an Fc domain of a human IgG1 antibody, which further increased their potency in inhibiting CXCR4 signaling and HIV-1-entry [15]. Label-based cellular assays measuring functional downstream responses of GPCR activation, as well as ligand displacement assays have proven to be highly valuable for GPCR research and drug discovery [16]. Real-time, label-free and cell-free ligand binding interactions are generally less considered in this area of research, mainly because of the difficulty of such assays due to the instability of GPCRs outside of their native cell membrane environment [17]. However, once optimized, label-free binding assays can provide useful information regarding interactions at the molecular level and accelerate target-specific drug discovery.

Surface plasmon resonance (SPR) sensors are real-time, label-free and often cell-free optical biosensors measuring interactions between molecules [18]. To investigate the binding properties of GPCR targeting molecules using SPR, synthetic peptides are often used to mimic certain epitopes of the receptors [19,20,21]. The downside of this approach is that the GPCR is not presented in its native conformation, and one cannot be certain that the binding behavior of designed epitopes are the same as that of analogous GPCRs. Therefore, studies have also been performed on full-length GPCRs, including rhodopsin [22], neurotensin [23], CXCR5 [24], CXCR4 [25,26,27] and CCR5 [26,28]. SPR-related full-length CXCR4 studies are most often performed in one of two following assay setups: either using virus-like particles (VLPs) [27,29,30,31] or using a C-terminal C9 peptide tag and an appurtenant 1D4 antibody surface [25,26,32]. The VLP setup guarantees the most stable state of the protein, however, the large size of the particles can exceed the 300 nm detection depth of the SPR sensor, resulting in a loss of binding detection [33]. The C-terminal C9 peptide tag offers a loose protein being stabilized by a detergent and lipid mixture, eliminating the size problem of VLPs. However, since this tag is not as widely used, expanding the assay to other membrane proteins may be more time consuming compared to a more commonly used affinity tag.

Here, we describe the development of an alternative SPR assay to characterize the binding kinetics of Nb-Fcs to full-length CXCR4 proteins immobilized on the chip by a C-terminal 10x-histidine tag. A carboxymethylated dextran pre-immobilized with a nitrilotriacetic acid (NTA) chip was used to successfully capture the histidine-tagged CXCR4. To the best of our knowledge, this is the first work reporting the use of a histidine tag to fix solubilized CXCR4 molecules on an NTA chip. Data from cell-based assays were used to validate the results obtained with this label-free assay.

## 2. Materials and Methods

### 2.1. Expression of CXCR4 in Sf9 Cells

Donor plasmid for full-length CXCR4 with inserted T4 lysozyme sequence was obtained from DNASU plasmid repository as described elsewhere [34]. High molecular weight recombinant bacmid containing the target gene sequence was prepared following the Bac-to-Bac Baculovirus Expression System manual (Invitrogen, Carlsbad, CA, USA). Sf9 cells (1 × 10^6^) were transfected by 6 μg bacmid using 8 μL CellFectin II reagent (Invitrogen, Carlsbad, CA, USA) in Sf-900 II serum free media (Life Technologies, Carlsbad, CA, USA) to generate recombinant baculovirus particles (P0 stock). Viral titer was increased by infecting 50 mL of sf9 cells at 1.2 × 10^6^ cells/mL density, as determined on LUNA-II Automated cell counter (Westburg, Leusden, The Netherlands), with 2 mL of the P0 stock and the suspension was allowed to grow for 72 h at 27 °C in a 250 mL shaking flask. Recombinant baculovirus particles were collected, filter sterilized and stored at 4 °C (P1 stock). For CXCR4 expression, sf9 cells at a density of 1.2 × 10^6^ cells/mL were infected with 3 mL of P1 virus and allowed to grow for 60 h when the cell viability dropped to 60–70%. Cells were harvested by centrifugation at 200× *g* and stored at −80 °C until further use. CXCR4 (over)expression on the sf9 cells was verified by flow cytometry according to Van Hout et al. using CXCR4-specific antibody clone 12G5 [14]. Both transfected and non-transfected sf9 cells (negative control) were analyzed.

### 2.2. Membrane Preparation and Solubilization

Insect cells (over)expressing CXCR4 on its membrane were disrupted in hypotonic buffer containing 20 mM HEPES pH 7.5, 20 mM KCl and 10 mM MgCl_2_ as described previously [34]. This was followed by Dounce homogenization (25 strokes) to separate membrane sheets from soluble fraction. The membrane fraction was collected by ultracentrifugation at 80,000× *g* followed by washing (3×) in a buffer containing 50 mM HEPES pH 7.5, 500 mM NaCl and 10% glycerol to separate soluble and membrane-associated proteins from integral transmembrane proteins. The membrane fraction was resuspended in solubilization buffer containing 20 mM Tris pH 7.0, 100 mM (NH_4_)_2_SO_4_, 10% glycerol, 0.07% cholesteryl hemisuccinate (CHS; Anatrace, Maumee, OH, USA), 0.33% n-dodecyl-β-d-maltopyranoside (DDM; Anatrace, Maumee, OH, USA), 0.33% 3-[(3-cholamidopropyl)-dimethylammonio]-1-propane sulfonate] (CHAPS; Anatrace, Maumee, OH, USA), 0.33 mM 1,2-dioleoyl-sn-glycero-3-phosphocholine/1,2-dioleoyl-sn-glycero-3-phospho-l-serine (DOPC:DOPS) mixed in a 7:3 *w*/*w* ratio (Avanti, Alabaster, AL, USA) and protease inhibitor tablet (Roche, Machelen, Belgium) [35]. The suspension was briefly sonicated and placed on a rocker for 3 h at 4 °C, after which the solubilized fraction was collected by centrifugation at 16,000× *g* for 30 min and stored at −80 °C until further use.

### 2.3. CXCR4 Immobilization and Stability Test

All SPR experiments were performed on a Biacore T200 (Cytiva, Marlborough, MA, USA) using carboxymethylated dextran chips pre-immobilized with NTA (Cytiva, Marlborough, MA, USA). The sample compartment temperature was kept at 15 °C and the analysis temperature at 25 °C for all SPR experiments. The running buffer used in the experiments was adapted from literature with slight modifications: 50 mM HEPES (pH 7.0), 0.1% DDM, 0.1% CHAPS, 0.02% CHS, 50 nM DOPC:DOPS (7:3) and 3 µM EDTA (running buffer 1) [26]. Immobilization of CXCR4 was performed using a standard nickel chelation procedure. First, the second channel of the chip was activated with a 60 s injection of 0.5 mM NiCl_2_ at a flow rate of 10 µL/min. Afterwards, the membrane fraction containing his-tagged CXCR4 diluted 1/3 in solubilization buffer was immobilized onto this channel by a 20 s injection at a flow rate of 10 µL/min. The capture level for all experiments averaged between 400–600 resonance units (RU). The first channel was not activated with nickel and was used as a reference surface.

To validate the stable conformation of CXCR4, conformation-dependent purified mouse anti-human CD184 (CXCR4) clone 12G5 (BD Biosciences, San Jose, CA, USA) was flown over both surface channels at concentrations ranging from 1.66–0.1 nM in a two-fold dilution step using running buffer 1. The monoclonal antibody (mAb) was injected at a flow rate of 30 µL/min for 120 s with a dissociation time of 600 s, using multiple cycle kinetics. The surface was regenerated using a 60 s injection of 350 mM EDTA followed by a 60 s injection of 1 M imidazole and finally by three 60 s injections of 50 mM NaOH. All regenerations were performed at 30 µL/min. Several buffer blanks were injected for double referencing. The experiment was performed in triplicate and was also repeated using 1D9 mAb (BD Biosciences, San Jose, CA, USA), as well as natural ligand CXCL12 (Peprotech, Rocky Hill, CT, USA), at the concentrations ranging from 13.3–0.42 nM and 50–3.125 nM, respectively, using two-fold dilution steps.

From the 12G5 mAb concentration series, 1.66 nM concentration was selected to test whether the mAb would bind to the protein when not solubilized in the stabilizing running buffer 1. For this purpose, the membrane fraction of CXCR4 overexpressing Sf9 cells was solubilized following the procedure as described above. However, here, we used a different buffer, which has the same ingredients as running buffer 1 except the detergent mix was replaced by 2% Triton X-100. The identical injection and regeneration procedures were followed as described above. The 12G5 mAb was injected at a 1.66 nM concentration over the surface of stabilized CXCR4, and after regeneration, this step was repeated using a non-stabilized protein of the same capture level. Buffer blanks were injected for double referencing.

### 2.4. Nanobody-Fc Binding to CXCR4 Using SPR

CXCR4 was immobilized as described in Section 2.3, and a period of 30 min was applied with only the running buffer flowing over the surface. This procedure was performed in order to ensure optimal surface stability by removing any unbound or weakly bound ligand as well as low affinity-bound background molecules. Nb-Fc constructs were produced in collaboration with research teams at VU Amsterdam and Argenx (Zwijnaarde, Belgium) as described previously [15]. The Nb-Fcs were diluted in running buffer 1. The Nb-Fc concentrations are all in a range between 10 nM–0.8 nM using two-fold dilution steps. The Nb-Fcs were injected at a flow rate of 30 µL/min for 120 s with a dissociation time of 600 s using multiple cycle kinetics. The surface was regenerated using a 60 s injection of 350 mM EDTA followed by a 60 s injection of 1 M imidazole and concluded by three 60 s injections of 50 mM NaOH. All regenerations were performed at 30 µL/min. Several buffer blanks were used for double referencing. The experiments were performed in triplicate. For the most potent Nb-Fc (VUN401-Fc), the binding results of both the bivalent Fc-version (VUN401-Fc) and monovalent version, without Fc domain (VUN401, QVQ), were obtained.

Since other molecules within the membrane fraction are known to have some nickel affinity [36], a negative control was set up to validate the binding results of the Nb-Fcs. Untransfected Sf9 cells not overexpressing CXCR4 (CXCR4-negative cells) were lysed and solubilized using the procedure as described in Section 2.2. The membrane fraction was injected over the activated NTA chip, and Nb-Fcs were injected using the same procedure as described above.

### 2.5. CXCR4 Virus-Like Particle Analysis Using SPR

For the most potent Nb-Fc (VUN401-Fc), the binding kinetics of both Nb constructs (VUN401 and VUN401-Fc) were also evaluated using human CXCR4 expressing biotinylated VLPs (Integral Molecular, Philadelphia, PA, USA). An SPR assay using CXCR4 VLPs is already described in the literature and thus offers a comparison basis for the assay developed in this study [31]. The CXCR4 VLP stock solution (4.3 units/µL) was diluted to 0.043 units/µL in running buffer 2 (0.01 M HEPES pH 7.4, 0.15 M NaCl, 1 mg/mL BSA) and immobilized on a streptavidin (SA) chip (Cytiva, Marlborough, MA, USA) at a flow rate of 1 µL/min for about 15 min until 900 RU were reached. Both Nb and Nb-Fc were injected at a flow rate of 30 µL/min using single cycle kinetics. Samples were injected at five different concentrations for 120 s each, followed by a dissociation time of 600 s and then by a 20 s regeneration step of HCl pH 3.0. The Nb was tested at 0.09–1.5 nM and the Nb-Fc at 0.08–1.25 nM, both using two-fold dilution steps. Several buffer blanks were included for double referencing. The experiments were performed in triplicate.

### 2.6. Flow Cytometry

The flow cytometry data of some of the Nb-Fcs were obtained from previous experiments [15]. The rest of the Nb-Fcs were analyzed using the same procedure. Briefly, the CXCR4 affinity of the Nb-Fcs was obtained by performing a binding assay using Jurkat cells. Jurkat cells (3 × 10^5^), resuspended in Dulbecco’s phosphate-buffered saline (DPBS) containing 2% fetal bovine serum (FBS), were treated with different concentrations of Nb-Fc (in DPBS/2% FBS) at RT for 30 min. Next, the binding of Nb-Fcs was detected with anti-human IgG Fc-γ-fragment-specific PE-conjugated Ab (polyclonal, ThermoFisher Scientific, Merelbeke, Belgium) at RT for 30 min. Cell suspensions were fixed in 1% paraformaldehyde (Merck, Darmstadt, Germany) in DPBS and analyzed with the FACSArray™ flow cytometer (Becton Dickinson, Erembodegem, Belgium).

### 2.7. Calcium Mobilization Assay

The calcium mobilization data of some of the Nb-Fcs were obtained from previous experiments [15]. The rest of the Nb-Fcs were analyzed using the same procedure. Briefly, 2 × 10^4^ cell/well U87.CD4.CXCR4 cells were seeded in gelatin-coated black-walled 96-well plates overnight at 37 °C and 5% CO_2_. Cells were loaded with 4 µM fluorescent calcium indicator Fluo-2 acetoxymethyl ester (Sigma-Aldrich, Saint-Louis, MO, USA) and treated with Nb-Fc constructs. Changes in intracellular calcium levels were measured by the FLIPR Tetra system after stimulating the treated cells with 6.25 nM CXCL12 (Peprotech, Rocky Hill, CT, USA).

### 2.8. Data Analysis

Apparent binding kinetics (K_D_, k_a_, k_d_) were derived after fitting the experimental data to the 1:1 Langmuir binding model using the Biacore T200 Evaluation Software 3.1. Data obtained from this software program were transferred to Graphpad Prism 8 for production of the sensorgrams. The sensorgrams start at 0 RU when the analyte is injected, the 400–600 RU immobilization response is automatically corrected to zero. Means and standard deviations were calculated using Excel. Simple linear regression of the correlation data was calculated using Graphad. Flow cytometry data were analyzed with the FlowJo^®^ Software (Ashland, OR, USA). The affinity (K_D_) derived from the flow cytometry data represents the Nb-Fc concentration needed to bind 50% of the CXCR4-expressing cells at equilibrium. The calcium mobilization data were analyzed with the ScreenWorks 4.0 software (Molecular Devices, Sunnyvale, CA, USA). Relative light units (RLUs) were corrected by subtracting the RLU measured at a specific time point just before CXCL12 addition from the RLUs measured on all other time points. Next, the difference between the maximum and minimum corrected RLUs was calculated.

## 3. Results

### 3.1. CXCR4 Expression Level in Sf9-Transfected Cells

**Figure 1:**
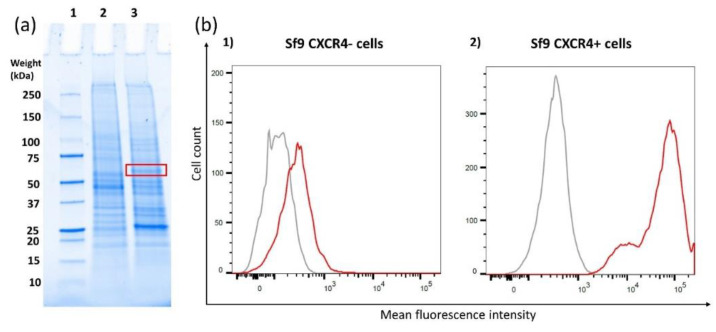
(**a**) SDS-PAGE of the membrane fractions of sf9-negative control cells (**a**, **2**) and sf9 cells overexpressing CXCR4 (**a**, **3**) including a control sample (**a**, **1**) indicating weight points. The red square indicates the band of CXCR4 which is only present in line 3. (**b**) Flow cytometry results depicting the binding of mAb 12G5 on sf9 cells in order to verify CXCR4 overexpression on the cell membrane. The left graph depicts the results from CXCR4-negative cells, which were not transfected with the CXCR4 plasmid (**b**, **1**). The right depicts the results the sf9 cells that were transfected with the CXCR4 plasmid (**b**, **2**). Cells stained with an isotype control Ab (IgG2a) (grey) were included as control, and cells stained with 12G5 mAb are shown as the red curve.

**Figure 2:**
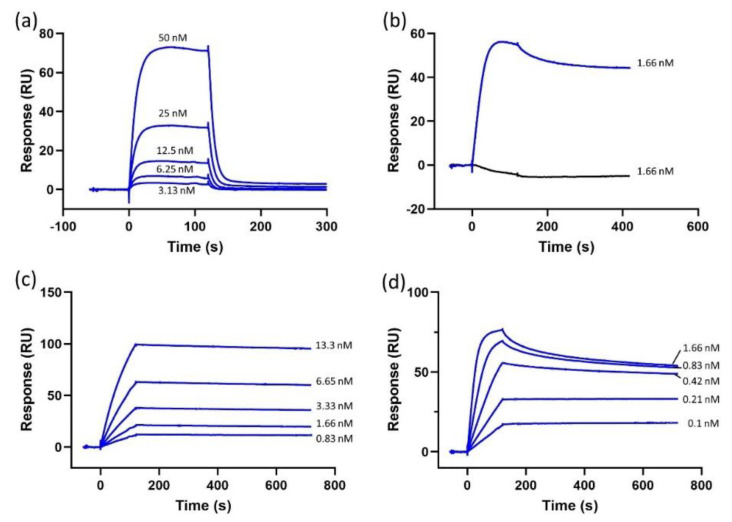
Sensorgrams depicting (**a**) CXCL12 (50, 25, 12.5, 6.25 and 3.13 nM) dose-dependent binding to histidine-tagged CXCR4 immobilized on a nitrilotriacetic acid (NTA) chip surface. (**b**) 12G5 mAb (1.66 nM) binding to histidine-tagged CXCR4 solubilized in regular solubilization buffer (blue) and the solubilization buffer with Triton X-100 (black). (**c**) 1D9 mAb (13.3, 6.65, 3.33, 1.66 and 0.83 nM) dose-dependent binding to CXCR4. (**d**) 12G5 mAb (1.66, 0.83, 0.42, 0.21 and 0.1 nM) dose-dependent binding to CXCR4.

**Figure 3:**
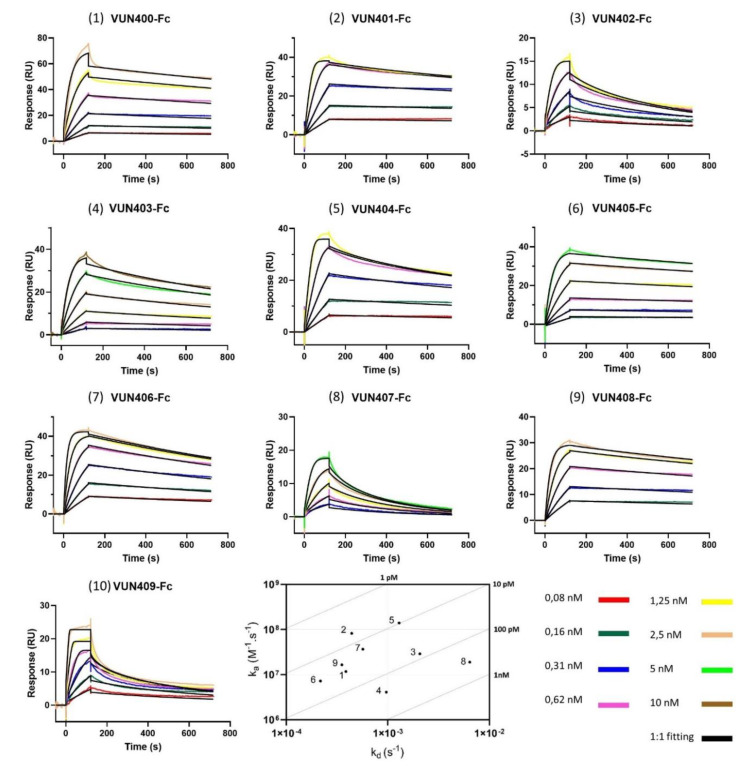
Sensorgrams of the binding curves (colored lines) and the fitted data (black lines) of the ten different nanobody-Fcs (Nb-Fcs) binding to histidine-tagged CXCR4 immobilized on an NTA surface. Concentrations of the injections are given in the legend. All experiments were carried out on a Biacore T200, and the data were fitted to the 1:1 Langmuir model using Biacore T200 Evaluation Software 3.1. An on-off rate map is included depicting the kinetic results of all Nb-Fcs (excluding VUN409-Fc), the oblique lines indicate the iso-affinities (K_D_).

The red box in the gel picture (Figure 1a) indicates the region where the expressed CXCR4 is present. It is shown that in the control sample, CXCR4-negative cells (Figure 1a, 2), there is no band, while in the overexpressed sample (Figure 1a, 3) a band is observed. The expression level of CXCR4 on the transfected sf9 cells (CXCR4-positive cells) was compared to the expression level on CXCR4-negative cells using flow cytometry. With the transfected cells, a clear shift towards higher fluorescence intensity is observed. This result demonstrates successful CXCR4 overexpression on the sf9 cells.

### 3.2. CXCR4 Immobilization

CXCR4 was captured on a Ni-NTA chip by means of a 10x-histidine tag fused to the C-terminal part of CXCR4. Once an NTA chip is loaded with Ni, it has the ability to capture and immobilize histidine-tagged proteins. Histidine tag–NTA binding is known to have less specificity compared to other tags, such as GST-tag. However, the histidine tag is cheap and widely available. Due to the lower affinity of the Ni-NTA surface towards histidine-tagged molecules, one cannot immobilize excessive amount of ligand since it will increase the chance of ligand leaching. This may lead to a decrease in ligand due to histidine tags being released from the NTA surface resulting in an unstable baseline [37]. Therefore, a period of 30 min between the capture of CXCR4 and injection of Nb-Fc was implemented to ensure optimal baseline stability. During this time, the running buffer flows over the surface. At a low capture level (not exceeding 600 RU), this method resulted in a baseline with acceptable stability (less than 0.05 RU/s decline). Zero-injections used for double referencing assured the corrections of any leftover baseline drift. Successful capture of CXCR4 showed that the histidine tag was accessible and can be used for immobilization.

### 3.3. CXCR4 Functionality

To evaluate CXCR4 protein functionality, we tested the binding of its natural ligand CXCL12 and two monoclonal CXCR4-targeting Abs. Dose-dependent binding response curves were obtained for CXCL12, 1D9 mAb and 12G5 mAb (Figure 2). The binding of conformation-dependent 12G5 mAb was important, since its binding can only occur when CXCR4 is in a stable conformation [38]. To further confirm the stable conformation, 12G5 mAb binding was tested against CXCR4 diluted in the running buffer 1 (i.e., mimicking its native environment) and in a denaturing buffer (i.e., an unstable environment). Substituting a DDM/CHS/CHAPS mix with Triton X-100 has already been proven to be a suitable method for testing the stability of CXCR4 [35]. The results (Figure 2b) confirm that CXCR4 is unstable in the Triton X-100-supplemented buffer as there is no response to the injected 12G5 mAb, demonstrating that 12G5 mAb does not bind to CXCR4 when the protein is not in its stable active conformation. In contrast, CXCR4 solubilized in DDM/CHS/CHAPS-supplemented buffer shows a good response resulting in the conclusion that the protein is functional and stable on the chip.

### 3.4. CXCR4 Nb-Fc Binding Using SPR

After confirming the CXCR4 was functional and stable, the next step was investigating the kinetics between CXCR4 and ten different Nb-Fcs. Binding responses for the ten Nb-Fcs are shown as dose-dependent curves in Figure 3. The corresponding binding rates are listed in Table 1. It is important to note that the association rate constant (k_a_) of Nb-Fc VUN409-Fc (10) was outside of the limits of reliable measurement by the biosensor, hence, this Nb-Fc is left out in further analysis. The sensorgrams show some striking differences in binding behavior. VUN407-Fc (8) and VUN402-Fc (3) seem to be less sticky compared to the other Nb-Fcs, which is shown by their relatively higher dissociations rate constants. This is also observed in their sensorgrams showing a more pronounced decline during the dissociation phase. VUN401-Fc (2) outperformed all other Nb-Fcs in terms of the binding affinity (K_D_), although it did not have either the highest association (k_a_) or lowest dissociation (k_d_) rate constant of all the Nb-Fcs. The combination of both a relatively high k_a_ and a low k_d_, however, resulted in the lowest K_D_ for this Nb-Fc. Another interesting result is shown between VUN403-Fc (4) and VUN407-Fc (8). Both have similar affinities, but they differ significantly in terms of association and dissociation constants. All Nb-Fcs show affinity in the picomolar range, however, these values are apparent affinities since they were calculated using the 1:1 Langmuir model. Due to possible avidity and rebinding effects of the Nb-Fcs, the binding behavior may deviate from a one-to-one binding model. Varying the immobilization density within the range of 300 to 700 RU has already shown to have no significant impact on the avidity [16]. Avidity and/or rebinding effects can result in slower dissociation rate constants compared to the perfect 1-to-1 binding values, hence, these values have to be interpreted with caution. Nevertheless, it can be concluded from our data that all tested CXCR4-specific Nb-Fcs are potent binders. Even though their affinities are quite similar, detailed kinetic information on these Nb-Fcs showed that they do behave different in terms of association and dissociation. This difference can be attributed to their binding behaviors. Van Hout et al. analyzed the binding behavior of three Nb-Fcs using shotgun mutagenesis epitope mapping and showed that these different Nb-Fcs bind to distinct epitopes of the CXCR4 receptor [14].

The immobilization of CXCR4 onto the NTA chip was not performed with a highly purified protein fraction but with the entire membrane fraction of the sf9 cells. As previously mentioned, it is known that certain proteins have histidine residues giving them some affinity towards this surface. There might be some non-specific proteins (background) captured onto the surface that could influence binding curves. In order to verify whether the binding curves did not show background binding, CXCR4-negative cells were used. These cells did not contain overexpressed histidine-tagged CXCR4. The cells were lysed, and the membrane fraction was solubilized in the exact same procedure as the cells overexpressing CXCR4. Afterwards, this CXCR4-negative membrane fraction was immobilized on a nickel activated NTA chip. A binding response, about 550 RU, was observed during the immobilization step (data not shown), confirming that this membrane fraction indeed contains residues with affinity towards nickel. Next, the ten Nb-Fcs were injected over the surface, each in three concentrations (0.63, 0.31 and 0.16 nM for VUN401-Fc, -404Fc, -406-Fc and -409-Fc; 1.25, 0.63 and 0.31 nM for VUN400-Fc, -402-Fc, 405-Fc and -408-Fc; and 2.5, 1.25 and 0.63 nM for VUN403-Fc and -407-Fc) by repeating the experimental procedure. No noticeable binding response was observed, except for a slight response for VUN400-Fc (1) and VUN402-Fc (3). However, they were not significant compared to the response using CXCR4-positive cells. These data indicate that the Nb-Fcs only bind to the CXCR4 present on the chip.

The experiments were repeated for the same Nb-Fc within 12 h using the same batch of CXCR4 (as well as at a later time with a different batch of CXCR4). The average drop in response between different runs, measured at the end of the association phase, was about 1.65 RU. This drop, allocated to both ligand leaching and protein decay, shows that both phenomena have negligible impact within the time range of conducting the experiment. The protein was stable for at least 12 h after being defrosted.

### 3.5. Comparison of the Monovalent and Bivalent Nanobody

**Figure 4:**
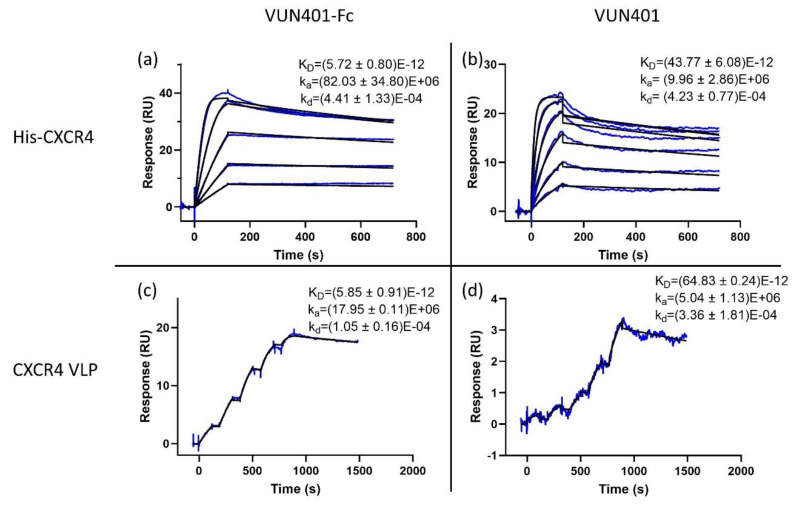
(**a**) VUN401-Fc bivalent Nb (concentration range of 1.25–0.08 nM using a two-fold dilution steps) binding to histidine-tagged CXCR4 immobilized on an NTA chip surface. (**b**) VUN401 monovalent Nb (concentration range of 12.5–0.39 nM using two fold dilution steps) binding to histidine-tagged CXCR4 immobilized on an NTA chip surface. (**c**) VUN401-Fc bivalent Nb (concentration range of 1.25–0.08 nM using two-fold dilution steps) binding to CXCR4 embedded in a virus-like particle (VLP). (**d**) VUN401 monovalent Nb (concentration range of 1.5–0.09 nM) binding to CXCR4 embedded in a VLP. K_D_ is expressed in M, k_a_ in M^−1^·s^−1^ and k_d_ in s^−1^.

The monovalent Nb VUN 401 consists of only one variable VHH domain. Consequently, this monovalent Nb has a lower molecular weight. Since SPR analysis is weight based, the concentration has to be slightly increased compared to its Nb-Fc counterpart in order to increase the response. In this experiment, the binding of the bivalent Nb-Fc was compared to its monovalent counterpart, and, in addition, binding affinities of Nb and Nb-Fc were also compared between our newly developed SPR assay and the established SPR assay using CXCR4 VLPs [31]. Both VUN401-Fc (Figure 4a,c) and VUN401 (Figure 4b,d) portray potent affinities (K_D_). The bivalent Nb-Fc outperformed the monovalent Nb by about a factor of 10. The difference is mostly from the higher (~10-fold) association rate constant of the bivalent Nb-Fc compared to the monovalent Nb, while both have very similar dissociation rate constants. When comparing our histidine-tagged CXCR4 assay to the CXCR4 VLP assay, only minor differences in kinetic rate constants are observed. When comparing both assay setups using the monovalent VUN401, the association and dissociation rate constants are very similar. However, when comparing the assays using VUN401-Fc, some differences in these constants are observed. This can be a result of the avidity of the Nb-Fc model. Since the monovalent Nb can only bind one ligand, its kinetics are closer to the theoretical 1:1 binding model, while the bivalent Nb-Fc has more chance of avidity effects. Since both assay setups are different, the effect of avidity can differ, which can result in dissimilarities in dissociation rate constants. A factor that could influence the association rate constant is the steric hindrance of other surface molecules on the VLP. Since the Nb-Fc is considerably larger than the Nb, it can be more prone to steric hindrance. However, the kinetic rate differences are within a reasonable range from each other, considering the fact that the two experimental setups are very different. The dissociation rate constant in our Nickel-NTA assay seems to correlate better between the mono- and bi-valent model compared to the CXCR4 VLP assay, suggesting that our histidine-tagged CXCR4 setup is less prone to avidity and/or rebinding effects.

### 3.6. Comparison of SPR Data with Cell-Based Assays

**Figure 5:**
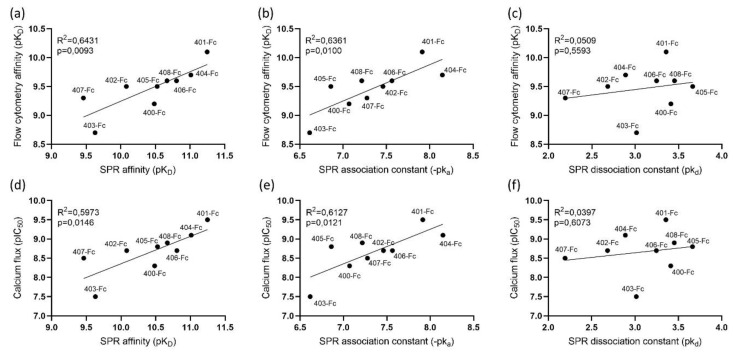
Correlation between surface plasmon resonance (SPR) affinity data with cell-based flow cytometry and calcium mobilization results. Comparisons between (**a**) the affinity (K_D_) measured by SPR and flow cytometry; (**b**) the SPR association rate constant (k_a_) and the flow cytometry affinity; (**c**) the SPR dissociation rate constant (k_d_) and the flow cytometry affinity; (**d**) calcium flux (pIC_50_) and the SPR affinity; (**e**) calcium flux and the SPR association rate constant; and (**f**) calcium flux and the SPR dissociation rate constant.

Figure 5 depicts the correlations between the obtained SPR results and the cell-based flow cytometry and calcium mobilization results. As shown in Section 3.4, VUN401-Fc outperforms all other Nb-Fcs in terms of affinity. This is in line with the results from the flow cytometry that also shows VUN401-Fc as the most potent binder. On average, the affinities obtained by SPR are a factor 10 higher compared to the ones obtained by flow cytometry. This difference could be attributed to the avidity or rebinding effects that might be more pronounced in one experimental setup compared to the other. Another possible explanation could be differences in the CXCR4 protein (e.g., T4 lysozyme sequence inserted in sf9 cells vs. native expressing CXCR4 expressed on Jurkat cells) and in the experimental environment. The SPR association rate constant as well as the SPR affinity correlate to the flow cytometry with about the same R^2^ (Figure 5a,b). The dissociation rate constant measured by SPR does not show any significant correlation with the affinity measured by flow cytometry (Figure 5c). This discrepancy might be a consequence of the kinetic influencing factors mentioned earlier. Comparison between the SPR results and the calcium mobilization assay (Figure 5d–f) shows about the same correlation as between SPR and flow cytometry. Both SPR affinity as well as association rate constants show correlation between the calcium flux (Figure 5d,e) and the SPR dissociation rate constant shows no correlation with the calcium flux (Figure 5f). Despite variability between the experimental environment and setup of the SPR assay and the cell-based binding assays (e.g., the different type of cells that were used and the environment the cells/protein was in), a correlation exists between both the affinity and association rate constant measured by SPR and the cell-based results.

## 4. Discussion

The ability to study chemokine receptors using biosensor technology contributes to a better understanding of the binding behaviors of these proteins. Several successful studies have been published involving membrane proteins using biosensors. However, in terms of chemokine receptors, the list is very concise. One study showed the potential of analyzing antibody interactions with histidine-tagged CXCR5 [24]; the receptor was expressed in sf9 cells using the FastBac expression system. We have successfully used the same expression system to express CXCR4, showing the potential of this system to expand to other chemokine receptors. The CXCR5 study not only included a capture stabilize step; the receptor protein was also purified. This approach is very useful in the investigation of the binding properties of small molecules, however, the assay is time consuming, requires well-purified protein, and has a higher risk of protein instability. In addition, in the case of protein purification, the potential effect on binding of interacting partners of the receptor that are present in the cell membrane is lost. In the case of GPCRs, this may be a relevant downside since interaction with other proteins (e.g., GPCR heterodimerization) is well established and can have great impact on ligand binding and receptor signaling. Both CXCR4 and CCR5 have also been used for biosensor studies using a C9-tag for immobilization purposes [26,28,32,35]. The advantages of the C9-tag over histidine tag is that the C9-tag is more specific, reducing the risk for high background signals by minimizing the binding of other molecules present in crude samples. Plasmids for histidine-tagged chemokine receptors, as well as other membrane proteins, are more widely and commercially available. Thereby, we tried to develop a simplified SPR assay setup using a histidine-tagged chemokine receptor. This ensures that expansion of this assay to other classes of chemokine receptors is more accessible. Our study has demonstrated and validated the potential of using an NTA surface to capture histidine-tagged CXCR4 to measure kinetics of receptor binding. The solubilized CXCR4 surface has been shown to be stable and active for at least 12 h. This SPR assay was thus created with the goal of reducing the experimental steps that can potentially impede the function of the chemokine receptor, leaving out protein purification and stabilization on the chip surface. This simplified assay should allow an easier transfer to other less explored chemokine receptors.

Our study validates, to our knowledge, the first Nb and Nb-Fc interaction with a solubilized GPCR in a real-time biosensor detection system. Nb and Nb-Fcs have shown great potential given their highly potent, specific and diverse modes of interaction with CXCR4 [14,15]. Our data elegantly demonstrate that Nbs and Nb-Fcs also have the potential to interact differently, in terms of binding kinetics, with their receptor. The Nb-Fcs used in this study have been developed with the aim of increasing the functionality of their smaller Nb formats [15]. The Nb-Fcs have shown to outperform their Nb counterpart in terms of binding affinity measured by flow cytometry, HIV replication inhibition, CXCR4 signaling inhibition, 125I-CXCL12 displacement and inhibition of CXCL12-induced G protein-mediated signaling. These characteristics give Nb-Fcs a great therapeutic potential. Additional information regarding the properties of Nb-Fcs can give extra validation to their potential. This makes biosensors a great addition to the cell-based assays, leading to a more in-depth understanding of real-time binding behavior. Our SPR results show that these Nb-Fcs have a very strong affinity towards CXCR4, validating their high potency observed in several cell-based assays. SPR results also showed that the Nb-Fc had a higher affinity compared to its monovalent counterpart, validating the increased potency of the Fc application model.

The assay was also compared with a CXCR4 VLP assay designed based on a published protocol [31]. The comparison showed comparable results validating that our assay produced reliable kinetic data. Interestingly, the dissociation rate constants for the Nb-Fc and the Nb were quite similar in our assay setup. These results could indicate that avidity might not be highly prevalent using our novel setup. This statement has to be treated with caution, as there was only one monovalent Nb available for this study, as well as the fact that rebinding can still occur with monovalent molecules. One of the advantages of our assay compared to the VLP setup is the ability to remove the entire surface during regeneration. VLPs are often immobilized on the surface in a non-reversible way; this could induce issues when using strong affinity binders. The Nb-Fc-CXCR4 complex is very strong and difficult to regenerate. Harsh regeneration conditions could affect the stability of the VLPs. Our setup eliminates the step to find applicable regeneration conditions. By renewing the CXCR4 surface with every injection, we ensure surface stability and eliminate the challenge of detaching strong complexes.

One of the main challenges in biosensor-based assays involving membrane proteins is retaining protein functionality as if it was inside the cell membrane. The correlation data shown in Section 3.6 indicate that even though CXCR4 was obtained from a different cell type and placed in a physiologically different environment, receptor functionality in the SPR assay and the effect of Nb-Fc correlated well with the data obtained from the cell-based flow cytometry assay. It must be noted that the kinetic results are apparent, as they are calculated using a 1:1 model, and the analytes are bivalent. In this case, it resulted in a dissociation rate constant that does not correlate with the affinity obtained from flow cytometry. Since the three kinetic parameters obtained by SPR are related to each other, the results from the affinity and the association rate constant of SPR do not rank the Nb-Fc potency in the same order. The assay in this study was created with the goal of having less steps in between that can protect the protein from potential damage. Importantly, elimination of assay steps that might damage the protein provides a higher probability that candidate for potency studies regarding Nb-Fcs. This assay could be used as a precursor to cell-based assays, picking only a selection of potent Nb-Fcs from the results and subsequently eliminating extra time in the cell-based assays that follow.

In conclusion, this study has confirmed the binding potency of CXCR4-specific Nb-Fcs as well as the potential for a biosensor-based chemokine receptor assay using histidine-tagged proteins and NTA chips. Our future studies will focus on expanding this assay to other CXCR4 ligands such as peptides or small molecules as well as expansion to other classes of histidine-tagged chemokine receptors.

## Figures and Tables

**Table 1:** Affinity (K_D_), association rate constant (k_a_) and dissociation rate constant (k_d_) of the ten Nb-Fcs calculated using the 1:1 Langmuir model.

	Affinity K_D_ (pM)	Association Rate Constant k_a_ (10^6^) (M^−1^·s^−1^)	Dissociation Rate Constant k_d_ (10^−4^) (s^−1^)
VUN400-Fc	33.57 ± 7.37	11.7 ± 1.16	3.87 ± 0.53
VUN401-Fc	5.72 ± 0.80	82.03 ± 34.80	4.41 ± 1.33
VUN402-Fc	87.97 ± 26.84	28.87 ± 17.43	20.73 ± 6.74
VUN403-Fc	236.33 ± 26.89	4.12 ± 0.47	9.66 ± 1.12
VUN404-Fc	9.82 ± 1.41	139.83 ± 74.57	12.92 ± 5.22
VUN405-Fc	30.27 ± 5.52	7.23 ± 0.73	2.17 ± 0.35
VUN406-Fc	16.03 ± 3.51	36.67 ± 11.69	5.68 ± 1.34
VUN407-Fc	353.67 ± 80.18	18.87 ± 4.21	64.23 ± 8.67
VUN408-Fc	21.70 ± 2.63	16.47 ± 2.15	3.53 ± 0.21

Mean and SD of three replicate experiments are shown.
